# The Effectiveness of Hyperbaric Oxygen Treatment in Patients with Complex Regional Pain Syndrome: A Retrospective Case series

**DOI:** 10.7150/ijms.97513

**Published:** 2024-08-01

**Authors:** Michal Hájek, Dittmar Chmelař, Jakub Tlapák, Miloslav Klugar

**Affiliations:** 1 Centre of Hyperbaric Medicine, Ostrava City Hospital, Ostrava, Czech Republic.; 2 Institute of Laboratory Medicine, Institute of Microbiology, Faculty of Medicine, University of Ostrava, Ostrava, Czech Republic.; 3 Centre for Hyperbaric Medicine of Faculty of Medicine University of Ostrava and Ostrava City Hospital, Ostrava, Czech Republic.; 4 Institute of Aviation Medicine, Prague, Czech Republic.; 5 Faculty of Biomedical Engineering, Czech Technical University in Prague, Czech Republic.; 6 Cochrane Czech Republic, The Czech Republic: JBI Centre of Excellence, Czech GRADE Network, Institute of Health Information and Statistics of the Czech Republic, Prague, Czech Republic.

**Keywords:** complex regional pain syndrome, hyperbaric oxygen therapy, pain, visual analog scale, case series

## Abstract

**Background:** Complex regional pain syndrome (CRPS) presents as persistent regional pain, both spontaneous and triggered. The demand persists for innovative treatments that patients can endure with minimal adverse effects. Hyperbaric oxygen therapy (HBOT) emerges as a possible intervention in this regard.

**Methods:** The main objective of this work is to retrospectively analyse a case series of patients diagnosed with CRPS treated in the Centre of Hyperbaric Medicine Ostrava over two years (period 2018-2019). The HBOT was applied at 2.0-2.4 absolute atmosphere (ATA) once a day.

**Results:** A total of 83 patients with CRPS were treated with HBOT. 98% of cases reported pain, 92% reported limitation of movement of the affected limb, 87% had swelling of the limb, 41% had lividity and 70% had sensory problems. The mean number of HBOT exposures was 22.0 ± 7.1. At the end of HBOT treatment, 86% of cases had symptoms relief. The mean VAS value of pain at rest before the start of HBOT was 3.2±3.0, after treatment it was 1.6±1.9 (p<0.001). In a pain at activity it was 6.1±2.4 and 3.7±2.4 (p<0.001), respectively, at the end of HBOT. The value of the functional assessment of the limb was 7.0±2.0 and 4.3±2.4 (p<0.001), respectively, at the end of treatment. 79 cases were included in the end-of-treatment assessment. 23 cases (29%) were evaluated as large clinically significant response, 48 cases (61%) were evaluated as partial response with minimally important difference. The results showed larger clinical HBOT effect in cases of disease duration up to 3 and 6 months (p=0.029).

**Conclusions:** The majority of patients improved pain and functional state of the affected limb. Our data also suggests the sooner after diagnosis of CRPS is HBOT started, the treatment has larger clinical effect. There was no serious HBOT-related complication or injury.

## Introduction

Chronic pain is among the most prevalent conditions in clinical practice. Various ailments, such as neuropathic pain, complex regional pain syndrome, migraine, and fibromyalgia, are related to the etiology of chronic pain 1-3. Complex regional pain syndrome (CRPS) is characterised by persistent regional pain, both spontaneous and triggered, typically originating in the distal limb. This pain exhibits an intensity or duration disproportionate to the expected course following similar tissue trauma 4. The condition involves multiple peripheral and central mechanisms, with specific factors exerting varying degrees of influence over time. These factors include musculoskeletal, peripheral, and central sensitization, autonomic alterations and sympathoafferent coupling, changes in receptor populations (such as adrenoreceptor upregulation and decreased skin nerve fiber density), cerebral alterations, genetic predispositions, psychological elements, inflammatory and immune responses, and central modifications in autonomic drive. These factors collectively contribute to disturbances in both regional and systemic sympathetic activity 4-6.

The estimated population incidence of CRPS stands at around 26 per 100,000 person-years, although this rate significantly escalates in certain contexts. Among the general populace, CRPS predominantly arises subsequent to fractures (occurring in over 40% of cases). In particular, prospective clinical investigations reveal a 31% incidence of CRPS after distal tibia fractures and an average occurrence of 18.8% after distal radius fractures. Other precipitating factors for CRPS include surgical procedures on hands and feet, sports-related injuries, and workplace accidents 7-9. Traditionally, CRPS is categorized into Type I and Type II, with the latter being less common and characterized by a documented nerve lesion. CRPS predominantly affects the upper extremities, with the highest incidence observed between 50 and 70 years of age 7. Patients with CRPS typically transition from an acute stage marked by pain, warmth, and edema in the affected limb to a chronic stage characterized by persistent pain despite the resolution of warmth and edema 4, 9.

CRPS exhibits a greater likelihood of responding to comprehensive multidisciplinary treatments that include medical, psychological, physical, and occupational therapies 1. Managing chronic pain, including CRPS, presents a formidable challenge that requires a multidisciplinary approach. Presently, the majority of pharmacological, non-pharmacological and interventional methods deliver only temporary or moderate relief from pain symptoms, often accompanied by intolerable side effects that compromise quality of life and result in poor adherence to treatment regimens. A systematic overview of reviews conducted within the Cochrane collaboration underscored the lack of high-quality evidence supporting the effectiveness of most therapies for CRPS. Specifically, limited evidence was found on the efficacy of bisphosphonates, calcitonin, intravenous ketamine, graded motor imagery (GMI) programs, and mirror therapy 10.

As per a recent review, contemporary CRPS treatment emphasizes physiotherapy and occupational therapy aimed at enhancing sensory perception, strength, motor skills (including fine motor skills), and sensorimotor integration/body perception 11. This approach is complemented by progressive psychological interventions to alleviate anxiety and avoidance behaviors, medications to alleviate inflammation and pain, passive physical techniques to reduce edema and pain, and assistive devices to improve daily functioning. Interventional procedures should be reserved for exceptional cases and conducted solely in specialized centers. Among these procedures, spinal cord and dorsal root ganglion stimulation demonstrate the most robust evidence. Modern diagnostic and treatment principles for CRPS integrate both physiological and psychological mechanisms, with the primary objective of restoring function and participation. However, further research is imperative to fortify the evidence base in this domain 11.

There is a pressing demand for novel chronic pain treatments that offer lasting relief without imposing significant side effects on patients. Hyperbaric oxygen therapy (HBOT) appears to be a promising addition to this treatment landscape. A growing body of evidence indicates that HBOT, characterized by its noninvasive nature and sustained efficacy, presents minimal side effects, positioning it as a viable option to address chronic pain conditions 12-15. HBOT has already gained prominence as the standard therapeutic approach to managing infected wounds, particularly those presenting with deep and chronic infections such as necrotizing fasciitis, osteomyelitis, chronic soft tissue infections, and diabetic foot infections 16, 17.

HBOT leads to a notable increase in tissue oxygen concentration, thus triggering neovascularization and angiogenesis, restoring tissue equilibrium, and increasing leukocyte function. Recent animal and human studies have indicated that HBOT induces analgesic effects across nociceptive, inflammatory, and neuropathic pain models, suggesting its potential utility in the treatment of various chronic pain syndromes, although the precise mechanism remains unclear 12. HBOT significantly increases the oxygen concentration in plasma, decreasing the dependence of hemoglobin for blood oxygen transport and exerting bacteriostatic or bactericidal effects, along with positive effects on the physiology of ischemic tissue after trauma or infection. Enhanced tissue oxygenation fosters fibroblast growth, collagen formation, angiogenesis, and the phagocytic activity of hypoxic leukocytes, thus reducing edema and facilitating tissue healing. Animal models of chronic, neuropathic, and inflammatory pain have demonstrated the antinociceptive and analgesic effects of HBOT. Human studies have further corroborated these findings, revealing the beneficial effects of HBOT on clinical outcomes such as pain scores, pain-related symptoms, and quality of life. Establishing a systematic approach to HBOT application is essential to validate its safety and efficacy 18.

The potential mechanisms underlying the positive effects of HBOT on CRPS are diverse and relate to the currently recognized pathophysiological causes of the condition. HBOT may exert its beneficial effects by restoring aerobic metabolism, correcting hypoxia and acidosis, and modulating nitric oxide (NO) activity and oxidative stress 19. Previous animal studies have demonstrated the analgesic properties of HBOT in models of nociceptive, inflammatory, and neuropathic pain 20,21. HBOT has been shown to alleviate mechanical hyperalgesia and inflammation in rodent models, with antinociceptive effects observed immediately after treatment and persisting up to 5 hours post-treatment 19. In patients with fibromyalgia syndrome (FMS), there is evidence suggesting that HBOT can alter brain metabolism and glial function, potentially rectifying abnormal brain activity associated with FMS 22. Additionally, research suggests that HBOT can induce neuroplasticity, leading to the restoration of impaired brain function and improved quality of life in post-stroke patients and those with prolonged post-concussion syndrome 23-25.

Data from Parkinson's disease models indicate that HBOT may play a neuroprotective role by reducing oxidative stress, neurodegeneration, and neuronal apoptosis 26. HBOT's significant anti-inflammatory effects across various conditions further suggest its potential to attenuate pain through the down-regulation of glial cell inflammatory mediators 27-30.

Unfortunately, over the last 2 decades, the amount of literature related to the use of HBOT in CRPS has been very limited. A total of six case reports and one RCT described the effects of HBOT in patients with CRPS 18, 31, 32. In review papers that describe treatment options in patients with CRPS HBOT is not mentioned very often. HBOT is mentioned more frequently in publications describing the problem of chronic pain or fibromyalgic syndrome (FMS).

A double-blind, randomized, placebo-controlled study with a limited sample size was conducted to evaluate whether HBOT was more effective than placebo in treating patients with post-traumatic CRPS of the wrist 32. Seventy-one patients were randomly assigned to either a treatment group (n = 37) receiving fifteen HBOT sessions at 243.1 kPa (2.4 atmospheres absolute) daily for 90 minutes, or a control group (n = 34) receiving fifteen daily 90-minute sessions in the hyperbaric chamber (also at 243.1 kPa) while breathing normal air.

After final treatment, patients with CRPS who received HBOT demonstrated significantly lower (improved) VAS scores, improved wrist extension, and reduced wrist edema compared to the control group 32. However, these results may be subject to potential confounding factors because the sham therapy chosen likely also had a positive, but smaller, effect on pain reduction 33.

## Materials and Methods

The main objective of this work is retrospective analysis of the case series of patients diagnosed with CRPS treated in the Centre of Hyperbaric Medicine Ostrava over two years. Secondary objective is to review current knowledge about CRPS with emphasis on pathophysiology, incidence and treatment.

The PROCESS reporting guideline was followed in the design and reporting of this retrospective single-centre consecutively collected case series 34.

### Patients

Patients have been primarily treated at various outpatient clinics and workplaces in the Moravian-Silesian, Olomouc, South Moravian and Zlín regions in the Czech Republic. After a referral phone call and consultation regarding the suitability of the indication and the occurrence or exclusion of contraindications for HBOT in a given patient between the treating specialist and the physician of the hyperbaric center, logistics were subsequently agreed and specified and the patient received the exact date of start of HBOT. All suggested patients without any restrictions were consecutively admitted to our centre.

All patients who underwent HBOT treatment in the Centre of Hyperbaric Medicine Ostrava over 2 years (in the period 2018-2019) were evaluated. Only four cases were excluded from the final assessment of outcome of HBOT as treatment had been ceased prematurely. All other data and results were included in the processing and analysis. An analysis of the medical records of all patients was performed and demographic data (age, sex), history, associated diseases, occupational and environmental influences, habits, especially smoking, referral specialist (surgeon, orthopaedic surgeon, neurologist, physiotherapist, etc.), type of injury, site of injury, diagnostic methods (X-ray, ultrasound, EMG, MRI, CT), signs and symptoms, degree of pain and functional impairment or limitation on the visual analogue scale (VAS) before and after the procedure were performed.

### HBOT Treatment

HBOT was carried out in a multiplace hyperbaric chamber for 10 patients (Vítkovice Steel company, Czechia, renovated by Haux-Life-Support, Germany). The hyperbaric chamber was equipped with a device for monitoring vital functions, a syringe pumps and Siaretron 1000 pulmonary ventilator device. Clinical evaluation by ENT specialist was conducted prior to treatment initiation, which included an evaluation of the ability to compensate pressure changes in the middle ear cavities. All patients were initially evaluated by physician of HBOT unit and signed an informed consent form. On request, some selected patients with predisposing conditions could have additional examinations performed prior to the start of HBOT, such as a lung examination by a specialist, chest X-ray, spirometry, etc. Just before the first exposure, patients were given medications with decongestant effect, usually nose drops or tablets orally. In case of difficulties during hyperbaric chamber compression with equalisation of pressures in the middle ear or other cavities in the head or face area, they could receive them at other exposures. In case of persistent difficulties, patients were referred to an ENT specialist for consultation, which is standard operating procedure. All patients were accompanied during treatment in the hyperbaric chamber by the staff of the Centre of Hyperbaric Medicine Ostrava, usually nurses.

HBOT was applied at 200-240 kPa, or 2.0-2.4 absolute atmosphere (ATA) once a day. Oxygen breathing lasted 80-90 minutes, with one 5 minutes air break included. The compression and decompression rates ranged from 6-10 kPa/min. Each patient had a minimum number of 20-25 exposures scheduled at the start of treatment. The total number of exposures then depended on the course of treatment, the tolerance of treatment, the development of the general condition as well as the local finding, the occurrence of events and adverse effects, as well as the individual needs and wishes of the patients. The course of HBOT treatment (time from onset of disease to initiation of treatment, treatment regimen used, number of HBOT exposures, medication used including analgesics) was evaluated.

Follow up: Long-term follow-up of patients after treatment is routinely not included in our centre's standard operating procedures, especially for capacity, operational and personnel reasons, nor has it been performed in this patient population.

### Adverse Effects

All events, adverse effects that occurred during treatment and hyperbaric chamber stay at any stage were evaluated, especially those that required treatment or therapeutic intervention (some medications including sedation, modification of treatment regimen, change in therapeutic pressure, rate of pressure changes, interruption or discontinuation of treatment etc.) and they are reported.

### Assessment of Pain and Functional Status

Patients were asked to evaluate the pain intensity on the VAS scale under rest conditions and during activity (walking if the disability site was on the lower limb, or during normal hand movements, grasping an object, etc. if the disability was on the upper limb). Similarly, the functional state of the affected limb, disability, the degree of limitation in normal activities such as walking, grasping objects, ability to write, comb hair, etc. was evaluated. This was performed twice, at the beginning and end of HBOT treatment. The VAS scale was measured by nurse during HBOT session in hyperbaric chamber or by physician during initial or final examination of patients.

### Relief of Symptoms

All patients were evaluated by physician during final examination (at the end of treatment) for relief (improvement) of symptoms during the entire treatment stay. It is a complex matter, a combination of an interview with the patient, who himself communicates what has changed, improved (e.g. some patients have some symptoms, swelling, lividity at certain times of the day, in the evening or in the afternoon, after exertion, long standing, walking etc, which is not possible to objectify at the time of examination), with an objective local examination of the affected limb and comparison with previous findings in the documentation. Photodocumentation of affected limbs was also carried out at least twice at the beginning and end, so overall it was possible to compare the extent of swelling, limitation of movement, colour and temperature changes on the limb and relief of symptoms during the entire treatment stay.

### Final Outcome of HBOT

The result of final outcome of HBOT was assessed by author team as a high clinically significant response (CSR) to treatment when symptoms were relieved and at the same time there was an improvement of at least 6 points on the VAS scale (sum of all 3 scales of rest pain, activity and functional status/limitations) and at the same time there was an improvement of 4 points or more on the functional scale. The functional state of the affected limb is subjectively reported by patients during the medical check before and after the treatment. Minimally important difference (MID) to treatment was assessed when symptoms were relieved and at the same time there was an improvement of at least 1 point on any VAS scale. The “overall response rate” is the summation of the CSR and MID. “No response” is used when the symptom does not show any improvement after therapy.

### Method of Processing and Analysis of Collected Data

Data on patients were obtained by detailed analysis of all medical files and by inclusion into the Microsoft Excel program (Microsoft Office Standard 2013). The above was done by one member of the team. None of the other members of the team participated in this activity. On the contrary, another member of the team was responsible for processing the supplied data and files into tables, statistical processing and writing the first draft of manuscript.

### Statistical Assessment

Mean values, standard deviation, median and quartiles values were used to describe demographic datasets, VAS scales, number of HBOT exposures and treatment outcomes. Differences in assessment of pain and functional state on VAS scales before and after HBOT therapy were assessed by the paired t-test. The final outcome of HBOT therapy in relation to disease duration were assessed by the chi-square test. P value less than 0.05 was considered statistically significant.

## Results

In 2018-2019, a total of 83 patients (36 patients in 2018, 47 in 2019) have been treated with hyperbaric oxygen in our unit, of which 27 (33%) men and 56 (67%) women. The mean age was 53.2 years (range 24-76). All patients were treated in an outpatient regimen, not one patient was hospitalised. Patients have been sent for treatment from outpatient workplaces of several specialisations, most often from orthopaedic (34 patients, 41%) and surgical (18, 22%) workplaces, as well as from neurology, plastic surgery, physiotherapy, algesiology and others. Regarding the location of the primary workplace of referral site workplaces from the whole Moravian-Silesian region dominate, only 5 (6%) patients were sent from other regions (South Moravia, Zlín region and Olomouc region).

### Demographic Data

Table 1 shows in detail all important patient demographic data, co-morbidities, harmful habits such as smoking, characteristics of injury or damaging event, and type of surgery as well. In 45 (54%) cases injury site was on the upper limb, in 38 (46%) cases on the lower limb. Regarding the type of injury, the most frequent were fractures in 37 (45%) cases. A large number of patients underwent surgery (51 cases, 61%) either in relation to the injury (osteosynthesis in 20 cases, external fixateur in 5 cases) or for another disease (arthroscopy of the knee, ligament surgery in 10 cases, carpal tunnel surgery in 9 cases), when the surgery very likely contributed to the development of CRPS. As regards the duration of symptoms and the disease, in 24 (29%) cases the duration was less than 3 months, in 24 (29%) cases a period of 3-6 months, in 20 (24%) cases 6-12 months and in 15 (18%) the duration of the difficulties was more than 1 year.

### Signs, Symptoms, Classification

A detailed assessment of signs and symptoms is listed in Table 2. It provides sensory, vasomotor, sudomotor and motor signs and symptoms evaluation. The most commonly observed symptoms were restriction of movement (range of motion) in 76 (92%) cases and swelling of the affected limb in 72 (87%) cases. Livid discolouration of the limb was among the most commonly observed symptoms in 34 (41%) cases as well as increased temperature of the affected limb (28 cases, 34%). Twenty-nine patients (35%) described reduced muscle strength. A total of 19 patients (23%) moved with the aid of crutches or French crutches. Budapest classification criteria were met in 74 (89%) cases. In terms of classification, 80 (96%) cases were classified as type I, and 3 cases as type II.

### Diagnostics and Treatment Details

The diagnosis was made using common diagnostic means. X-ray of the affected limb was the most common diagnostic tool, in 70 cases (84%). Signs of osteoporosis were the most common finding in 53 cases (64%). Additional imaging methods such as CT in 7 cases (8%) and MRI in 8 cases (10%) were also used. An overview of diagnostic methods is provided in Table 3.

All patients were treated commonly used therapeutic agents prior or during HBOT treatment, usually with a combination of pharmacotherapy and physiotherapy. The specific treatment of the patient was individualised and was the responsibility of the referring specialist. This involved a relatively large number of preparations from many drug groups. However, the most common treatments were calcium substitutes (22 cases, 27%), vitamin D3 (11 cases, 13%) or combinations thereof (23 cases, 28%). Additionally were further administered venotonics, ergot alkaloids, non-steroidal anti-inflammatory drugs (NSAID), benzodiazepines, anxiolytics, antidepressants, analgesics and anticonvulsants with an analgesic effect (pregabalin, gabapentin).

In addition to pharmacotherapy, patients underwent various physiotherapy and rehabilitation methods and techniques. Comprehensive rehabilitation was performed in 54 cases (65%). The most frequent treatment was an individual physiotherapy program in 24 cases (29%), soft tissue mobilisation therapy in 13 (16%) cases and magnetotherapy in 10 cases (12%). Details are listed in Table 4.

### Course of HBOT, Adverse Effects

83 patients were treated with HBOT at a pressure 2.0-2.4 ATA. A total of 1831 exposures were applied, of which 1195 were at a pressure of 2.0 ATA and 636 were at a pressure of 2.4 ATA. The mean number of exposures was 22.0 ± 7.1 (range 1-36, median 24, Q1 - 20, Q3 - 26).

Overall, 30 (36%) patients reported discomfort during treatment, most commonly discomfort in the ear or pain in the ear when chamber pressure was increased during compression (28 cases, 34%). In 2 cases there was psychological discomfort or claustrophobia from the confined space of the hyperbaric chamber. As the treatment lasted on average 22 days, there were other discomforts and obstacles that could lead to interruption of course of HBOT. Some of them were not related to the treatment itself (family or work reasons), but also were reported intercurrent seasonal illness (influenza). 13 patients (16%) have interrupted course of HBOT, most often for ear discomfort (5 cases). After the problem was solved, the problems subsided or the intercurrent disease was cured, they continued the treatment. All data and details of patients related to HBOT as well as all adverse effects are listed in Table 5.

Overall, 79 patients (95%) completed HBOT on schedule. Only four patients (5%) ceased HBOT, in 2 cases for ear discomfort and mediootitis, in 1 case for dental inflammation and in 1 case for claustrophobia. There was no serious HBOT-related complication or injury.

### Assessment of Pain and Functional State of Affected Limb

Total of 81 (98%) patients reported pain before HBOT. The mean VAS value of pain at rest before the start of HBOT was 3.2±3.0, after treatment it was 1.6±1.9. In a category pain at activity it was 6.1±2.4 and 3.7±2.4, respectively, at the end of HBOT. The value of the functional assessment of the limb was 7.0±2.0 and 4.3±2.4, respectively, at the end of treatment. The results were statistically significant (paired t-test) at the level of statistical significance p<0.001 for all three categories. Most importantly, the results are clinically significant for patients as from the pain so from the functional perspective. Detailed assessment is listed in Table 6.

### Relief of Symptoms

All patients were evaluated at the end of treatment by physician during final examination for relief (improvement) of symptoms during the entire treatment stay. It was found that in 71 of cases (86%) symptoms relieved, 12 cases (14%) had no symptom relief.

### Final Outcome of HBOT

Evaluation of final outcome of HBOT is listed in Table 7. In the final assessment of outcome of HBOT 4 cases were excluded as treatment had been ceased prematurely (cases described above who experienced difficulties with HBOT). Thus, 79 cases were included in the end-of-treatment assessment. 23 cases (29%) were evaluated as large clinically significant response (CSR), 48 cases (61%) were evaluated as partial response with minimally important difference (MID). Only 8 cases (10%) were evaluated as non-response. The “overall response rate” as the summation of the CSR and MID revealed 71 cases (90%).

### Final Outcome Related to Duration of the Disease

Final outcome related to duration of the disease (79 cases) is listed in Table 8. The data showed that there is a correlation between CSR and MID results in relation to the duration of symptoms. For example, there were 9 cases in the category of disease duration up to 3 months for the CSR result compared to 2 cases in the category of disease duration more than 12 months. Similarly, there were 15 cases in the category of disease duration up to 3 months for the MID result compared to 7 cases in the category of disease duration more than 12 months. The opposite correlation is in the category No response, where there was not a single case in the category of disease duration up to 3 months compared to 8 cases in the category of disease duration more than 12 months. The results were statistically significant (chi square test) at the level of statistical significance p=0.029.

## Discussion

Certain risk factors predispose people to develop complex regional pain syndrome (CRPS) after surgical treatment for traumatic hand injuries. In a study involving 291 patients with such injuries, CRPS was diagnosed in 68 patients (26.2%), with an average duration of 40.10 ± 17.01 days between surgery and CRPS diagnosis. CRPS-diagnosed patients experienced significantly higher levels of postoperative pain compared to those without CRPS (p < 0.001). Specifically, patients with a pain score of ≥5 within the first 3 days after surgery and those with crush injuries were identified as being at a heightened risk for CRPS development after surgical intervention for traumatic hand injuries 35. Another study aimed to assess the risk of developing CRPS in a new extremity after an initial diagnosis of CRPS in a different limb. It was found that 19 of 93 patients (20.4%) experienced CRPS in another extremity after further surgery or injury, a rate significantly higher than the general incidence reported in the existing literature (23.4 per 100,000; p < 0.0001) 36. Among twenty patients who experienced a documented secondary injury or surgery in a different extremity, fifteen patients (75%) developed secondary CRPS. This incidence significantly exceeds the reported CRPS incidence rate of 6.4% following distal radius fracture, as indicated by a review of the literature (p < 0.001, 95% CI 5.9-23.2) 36. These findings suggest that individuals with a history of CRPS are at a higher risk of developing secondary CRPS compared to the rates observed in the general population. Consequently, patients with a history of CRPS should be informed that they may face an increased risk of developing secondary CRPS if they undergo surgery or sustain trauma to another extremity 36.

HBOT is not standardized in the CRPS management, but all studies published on the topic, including our results, suggest it is a promising solution in both acute and chronic treatment of the disease. Due to the symptoms that limit patients in daily life, early diagnosis and active treatment approach directly after onset of CRPS are crucial factors that improve patient prognosis. Most of the patients in our retrospective analysis improved pain and functional state of the affected limb. Our data also suggests the sooner after diagnosis of CRPS (up to three months) is HBOT started, the treatment has larger clinical effect. Results until six months are also beneficial to patients, however, the effectiveness decreases over time in our sample (Table 8).

Despite some supportive evidence indicating a positive effect of HBOT on CRPS, this chronic pain condition is notably absent from the lists of approved indications by major professional societies, such as the 10th ECHM Consensus Conference in 2016 37. Furthermore, HBOT is often overlooked in recent review articles or systematic reviews, either not mentioned or excluded from the analysis, mainly due to its classification as a treatment method that is not commonly used 10, 38, 39, 40.

In our study, there were no serious complications or injury related to HBOT, which confirms previously published papers that HBOT is a safe treatment method.

### Limitations and Strengths of the Study

We are aware that this study has several methodological limitations, which are underlined by the study design which we had to choose. The retrospective case series descriptive observational study design. We were seeking in our country for the controlled group, however we failed to identify a larger sample. The sample size is also limited to the capacity of our Hyperbaric centre. On the other hand, we consecutively admitted all patients who were suggested to our centre without any pre-selection. Although our statistical analyses are performed on a small sample in the before and after data evaluation of one sample, the change in absolute numbers suggests a large potential of HBOT in the treatment of CRPS, which should be further studied with properly designed RCT. Data before and after the study were collected by an experienced trained nurse and data were analysed by another colleague, so we tried to limit some of the measurement biases.

## Conclusions

The majority of patients improved pain and functional state of the affected limb. Our data also suggests the sooner after diagnosis of CRPS is HBOT started, the treatment has larger clinical effect. There was no serious HBOT-related complication or injury, confirming previously published papers that HBOT is a safe treatment method. We are aware this study has several methodological limitations. Although our statistical analyses are performed on a small sample, the change in absolute numbers suggests a large potential of HBOT in the treatment of CRPS, which should be further studied with properly designed RCT.

### Recommendations for Further Research and Practice

Based on our literature review and our own results, we recommend for future research to design a multicentric double-blinded randomised controlled study with optimal information size, which includes patients ideally up to three months after the CRPS symptoms. Objective measurement of functional outcomes should be included and patients in both groups should have similar baseline characteristics, including their other treatments.

Implication for practice: The certainty of the existing evidence on the effectiveness of HBOT in CRPS patients is low, but it shows a large clinical effect on pain reduction and improvement of the functional state of the affected limb. Guideline developers should consider developing conditional recommendations for the use of HBOT in CRPS patients until the study with proper design and sample size is completed.

## Author contributions

M.H., M.K. participated in the research idea and in its design. M.H., D.CH., J.T., M.K. contributed to the analysis of articles for methods and results. M.H. led the study supervision. M.H., D.CH., J.T., M.K. contributed to the interpretation of the results. All authors have read and agreed to the published version of the manuscript.

## Figures and Tables

**Table 1:** Demographic data, co-morbidities, characteristics of injury, surgery and duration of disease

	N ^1^	% ^2^
Men/women	27/56	33/67
Age (years)	mean±SD ^3^ 53,2±11.1	median 54, range 24-76
**Co-morbidities**	**N ^1^**	**% ^2^**
Hypertension	28	34
Diabetes	4	5
Chronic respiratory disease, asthma, COPD	17	20
Thyroid disease	13	16
Rheumatoid arthritis	4	5
Chronic venous insufficiency	5	6
Arrhythmias	6	7
Psychiatric disorders	7	8
Without comorbidities	30	36
Smoking	15	18
**Occupation**	**N ^1^**	**% ^2^**
Employed	54	65
Unemployed	5	6
Retired	16	19
Disability pension	8	10
**Site of injury**	**N ^1^**	**% ^2^**
Upper extremities	45	54
Lower extremities	38	46
**Type of injury**	**N ^1^**	**% ^2^**
Fractures	37	45
Sprains, contusions	18	22
Meniscus knee injury	4	5
Tendon injury	5	6
Ligament injury	2	2
**Surgery**	**51**	**61**
Osteosynthesis	20	24
External fixateur	5	6
Arthroscopy of the knee, Ligament surgery	10	12
Surgery of the hand (carpal tunnel, Dupuytren's contracture etc)	12	14
Other	7	8
**Duration of the disease**	**N ^1^**	**% ^2^**
Less than 3 months	24	29
3-6 months	24	29
6-12 months	20	24
More than 12 months	15	18

^1^ N, number of clinical participants; ^2^ %, percentage representation of clinical participants; ^3^ SD, standard deviation

**Table 2:** Signs, symptoms

sensory	N (%) ^1,2^	vasomotor	N (%) ^1,2^	sudomotor	N (%) ^1,2^	motor	N (%) ^1,2^
Paraesthesia	15(18)	Lividity	34(41)	Swelling	72(87)	Range of motion restriction	76(92)
Tingling	11(13)	Increased temperature	28(34)	Sweating	7(8)	Grip limitation	19(23)
Burning	7(8)	Redness	17(20)			Fist fails to close	14(17)
Numbness	7(8)	Colour changes	15(18)			Stiffness	13(16)
Hyperaesthesia	11(13)	Cold	12(14)			Objects fall out	5(6)
Pulsating	3(4)	Shiny skin	4(5)			Can't make a pinch	3(4)
Pinching	2(2)	Whitish discoloration	2(2)			Tremor	1(1)
Neuropathy	2(2)	Paleness	2(2)				
Dysaesthesia	2(2)						

^1^ N, number of clinical participants; ^2^ %, percentage representation of clinical participants

**Table 3:** Diagnostics details

Diagnostics methods	N (%) ^1,2^				
X-ray	70(84)				
osteoporotic changes	53(64)				
other changes	9(11)				
EMG	14(17)				
MRI	8(10)				
CT	7(8)				

^1^ N, number of clinical participants; ^2^ %, percentage representation of clinical participants

**Table 4:** Treatment details

Pharmacotherapy	N (%) ^1,2^	Physiotherapy methods	N (%) ^1,2^
Calcium	22(27)	Comprehensive rehabilitation	54(65)
Calcium + vitamin D3	23(28)	Individual physiotherapy	24(29)
Vitamin D3	11(13)	Soft tissue mobilisation therapy	13(16)
Venotonics, venopharmaceuticals	24(29)	Magnetotherapy	10(12)
Ergot alkaloids	20(24)	Remedial gymnastics	8(10)
NSAID	18(22)	Ergotherapy	8(10)
Benzodiazepines, SSRIs, anxiolytics	18(22)	Electrotherapy	7(8)
Paracetamol, analgesics	15(18)	Jacuzzi	3(4)
Pregabalin, Gabapentin	12(14)	Swimming	2(2)
Vitamins E, B	7(8)		
Antihistamines (Prothazine)	6(7)		
Wobenzym	3(4)		
Bisoprol	3(4)		

^1^ N, number of clinical participants; ^2^ %, percentage representation of clinical participants

**Table 5:** Course of HBOT, adverse effects.

HBOT ^4^	2.0-2.4 ATA ^5^
Number of exposures	1831
mean±SD ^3^	22.0 ± 7.1
median	24
Q1	20
Q3	26
**Patients reported discomfort during HBOT ^4^**	**N (%) ^1,2^**
Total score	30 (36)
Ear discomfort	28 (34)
Psychical discomfort, claustrophobia	2 (2)
**Interruption of HBOT ^4^**	**N (%) ^1,2^**
Total score	13(16)
respiratory tract infection, influenza	2 (2)
work and family reasons	3(4)
ear discomfort, mediootitis	5(6)
Psychical discomfort, claustrophobia	1(1)
dental inflammation	1(1)
phlebothrombosis	1(1)

^1^ N, number of clinical participants; ^2^ %, percentage representation of clinical participants; ^3^ SD, standard deviation; ^4^ HBOT, hyperbaric oxygen treatment; ^5^ ATA, absolute atmosphere

**Table 6:** Assessment of pain and functional state of affected limb.

	Before HBOT ^2^ (baseline)				After HBOT^ 2^				Paired t-test
	mean±SD ^1^	median	Q1	Q3	mean±SD ^1^	median	Q1	Q3	
Pain at rest	3.2±3.0	3	0	5	1.6±1.9	1	0	3	p<0.001
Pain at activity	6.1±2,4	7	4	8	3.7±2,4	3.5	2	5	p<0.001
Functional state of affected limb	7.0±2.0	7	5	8	4.3±2.4	4	2.25	5	p<0.001

^1^ SD, standard deviation; ^2^ HBOT, hyperbaric oxygen treatment

**Table 7:** Final outcome of HBOT (79 cases).

Final outcome	N (%) ^1,2^
Large clinically significant response (CSR)	23 (29)
Minimally important difference (MID)	48 (61)
Overall response rate	71 (90)
No response	8 (10)

^1^ N, number of clinical participants; ^2^ %, percentage representation of clinical participants

**Table 8:** Final outcome and duration of the disease (79 cases).

p = 0,029 (chi square test)	up to 3 months	3-6 months	6-12 months	over 12 months	total
Large clinically significant response (CSR)	9	6	6	2	23
Minimally important difference (MID)	15	14	12	7	48
Overall response rate	24	20	18	9	71
No response	0	1	2	5	8

## Abbreviations

ATA: absolute atmosphere
CRPS: Complex regional pain syndrome
CRS: clinically significant response
ECHM: European Committee for Hyperbaric Medicine
FMS: fibromyalgia syndrome
GMI: graded motor imagery
HBOT: hyperbaric oxygen treatment/therapy
MID: minimally important difference
NSAID: non-steroidal anti-inflammatory drugs
RCT: randomized controlled trial
VAS: visual analogue scale
